# Extracellular Phytase Production by the Wine Yeast *S. cerevisiae* (Finarome Strain) during Submerged Fermentation

**DOI:** 10.3390/molecules23040848

**Published:** 2018-04-08

**Authors:** Grzegorz Kłosowski, Dawid Mikulski, Oliwia Jankowiak

**Affiliations:** Department of Biotechnology, Kazimierz Wielki University, ul. K. J. Poniatowskiego 12, 85-671 Bydgoszcz, Poland; klosowski@ukw.edu.pl (G.K.); ojankowiak@op.pl (O.J.)

**Keywords:** phytase, *S. cerevisiae*, catalytic activity, phytic acid utilization

## Abstract

One of the key steps in the production of phytases of microbial origin is selection of culture parameters, followed by isolation of the enzyme and evaluation of its catalytic activity. It was found that conditions for *S. cerevisiae* yeast culture, strain Finarome, giving the reduction in phytic acid concentration of more than 98% within 24 h of incubation were as follows: pH 5.5, 32 °C, continuous stirring at 80 rpm, the use of mannose as a carbon source and aspartic acid as a source of nitrogen. The highest catalytic activity of the isolated phytase was observed at 37 °C, pH 4.0 and using phytate as substrate at concentration of 5.0 mM. The presence of ethanol in the medium at a concentration of 12% *v*/*v* reduces the catalytic activity to above 60%. Properties of phytase derived from *S. cerevisiae* yeast culture, strain Finarome, indicate the possibility of its application in the form of a cell’s free crude protein isolate for the hydrolysis of phytic acid to improve the efficiency of alcoholic fermentation processes. Our results also suggest a possibility to use the strain under study to obtain a fusant derived with specialized distillery strains, capable of carrying out a highly efficient fermentation process combined with the utilization of phytates.

## 1. Introduction

Monogastric animals breeding (pigs and poultry production), conducted on an industrial scale, generates severe environmental pollution with phosphorus compounds, which may cause eutrophication of surface water. The presence of high concentrations of phosphorus compounds in livestock excreta is a result of the low availability of compounds of this element in plant feeds that are rich in phytic acid, which is the main form of phosphorus storage in cereals and legumes [1,2]. Phytic acid, occurring mainly in the form of complexes with divalent cations (Mg^2+^, Ca^2+^, Zn^2+^, Fe^2+^) and proteins, is not an assimilable form of phosphorus for organisms that are not capable of hydrolyzing these complexes, including monogastric animals, humans and selected strains of microorganisms [3].

Phytases are enzymes that allow hydrolysis of phytic acid complexes. These enzymes catalyze the release of orthophosphates and compounds bound in phytic complexes thereby increasing their bioavailability. Phytases are isolated from plant tissues (EC 3.1.3.26) as well as from microbial cells and post-culture media (EC 3.1.3.8). However, due to the higher catalytic activity, only microbial strains derived mainly from *Aspergillus* fungi [4] are used in industrial processes. The problem of phytic acid complexes is not confined to the feed industry, but it is also present in the distillery industry. A negative impact of phytic acid on the course and efficiency of the alcoholic fermentation of high gravity (HG) media was observed [5]. These media were prepared with the use of raw materials such as corn grain in which nearly 80% of phosphorus is bound in phytic acid [6].

The presence of factors limiting the availability of phosphorus compounds in raw materials, both in the feed and the distillery industries, leads to the search for new, more efficient sources of enzymes capable of hydrolyzing phytic acid. Numerous studies on phytases isolated from various groups of microorganisms, such as filamentous fungi, bacteria and yeast, have been conducted to characterize their catalytic activity and to select the culture conditions for microorganisms that would ensure the effective utilization of phytic acid. The use of cultures of various microorganisms for the hydrolysis of phytic acid comes from the possibility of application of the enzyme isolated from the post-culture medium, or the use of microorganisms producing highly effective phytases in situ during feed production or during fermentation processes. An important element in the development of biosynthesis of enzymatic proteins, including phytases, is the selection of suitable culture conditions such as: culture composition, temperature and pH, to achieve an efficient protein production process or to ensure a high degree of biotransformation of available substrates. Efficient phytase production requires precise development of culture parameters adapted to the requirements of a given group of microorganisms. It is important to choose appropriate sources of carbon and nitrogen, while indicating the most favorable temperature and pH conditions. Selection of culture parameters for efficient phytase production depends on preferences of particular species and even individual strains of microorganisms [7,8]. An increase in phytase production is observed in the *Pichia anomala* culture supplemented with additional nitrogen source. The addition of urea, ammonium nitrate, ammonium chloride and asparagine results in a doubling of phytase production, relative to the control medium [9]. In the cultivation of these yeasts, aimed at the efficient production of phytases, the presence of metal cations and a suitable carbon source is also important. An increased availability of iron and calcium ions intensifies the production of enzymatic proteins with hydrolytic activity against phytic acid, while the availability of lactose, xylose and starch as carbon source in *Pichia anomala* culture reduces the synthesis of phytases [10]. In the *Bacillus subtilis* US417 culture, a high yield of phytase production was achieved as a result of the selection of the culture medium parameters. The basic ingredient of the medium was wheat flour; yeast extract was used as a source of nitrogen and methanol was the carbon source [11]. Supplementation of culture media with tryptone, MgSO_4_, asparagine and a chemical surfactant (Tween-80) positively influenced phytase production by *Sporotrichum thermophile* [12]. A negative influence of urea on phytase biosynthesis was observed for the cultures of *S. cerevisiae*, in contrast to the cultures of *Aspergillus ficuum* and *Pichia anomala* [13]. The highest phytase activity in the culture of *S. cerevisiae* CY-3079 strain was observed when galactose was the carbon source and ammonium sulphate was used as a nitrogen source [14]. Phytase prepared from a culture requires a catalytic characterization that includes the influence of pH, temperature and the selected metal ions on its enzymatic activity. Different species and even strains of fungi, yeast and bacteria have their own requirements for pH or temperature [3,4,15].

The negative effect of phytic acid on yeast activity in the raw materials used to prepare the fermentation media has been an impulse for the search for *S. cerevisiae* yeast strains capable of utilizing phytic acid in situ. The aim of the study was to select the media composition such as carbon and nitrogen sources and the selection of culture parameters such as temperature and pH for the analyzed *S. cerevisiae* yeast strain Finarome as phytase producer. Preliminary characterization of catalytic activity of the isolated phytase was also performed to evaluate the usefulness of this enzyme in fermentation processes as a supportive enzyme. After screening yeast of the genus *Saccharomyces* [16], the experiments reported in this study constitute an important step toward the development of a *S. cerevisiae* yeast strain, via protoplast fusion, capable of carrying out a highly efficient alcoholic fermentation combined with the utilization of phytic acid.

## 2. Results and Discussion

### 2.1. Influence of Medium Components and Culture Conditions on the Degree of Utilization of Phytic Acid

**Figure 1:**
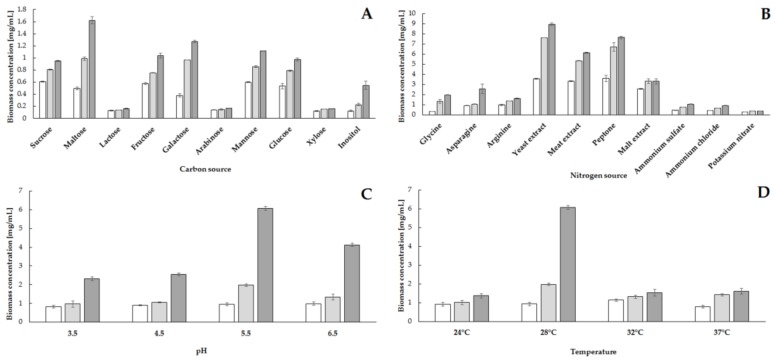
Influence of carbon source (**A**), nitrogen source (**B**), pH (**C**) and temperature (**D**) on growth rate of yeast biomass of *S. cerevisiae* strain Finarome during culture (24 h of culture—white bars, 48 h of culture—light gray bars, 72 h of dark gray bars).

**Figure 2:**
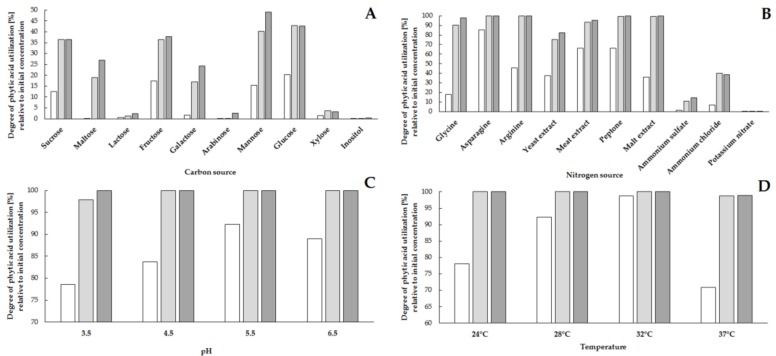
Influence of carbon source (**A**), nitrogen source (**B**), pH (**C**) and temperature (**D**) on the reduction of phytic acid concentration during culture of *S. cerevisiae* strain Finarome (24 h of culture—white bars, 48 h of culture—light gray bars, 72 h of dark gray bars).

The high growth rate of microbial biomass during culture and the production of enzyme proteins depend primarily on the availability of sources of carbon, nitrogen and growth factors, and the parameters of the culture process, i.e., temperature, pH and culture time. The proper selection of culture conditions guarantees a highly efficient production of biocatalysts and a high degree of bioconversion of the substrate using enzymatic proteins [7]. As a part of the selection of the culture process, the effects of various sources of carbon and nitrogen as well as temperature and pH on growth rate of *S. cerevisiae* biomass, strain Finarome, and the degree of utilization of phytic acid were determined based on the concentration of phytate in the culture, compared to the initial concentration (Figure 1 and Figure 2).

When analyzing the effect of carbon source, we found that the highest concentration of yeast biomass at 1.6 mg mL^−1^ after 72 h of culture was obtained in a medium containing maltose as a carbon source (Figure 1A). However, the tested strain also exhibited a high biomass production after 72 h of culture (about 1 mg mL^−1^) in media containing sucrose, fructose, galactose, mannose and glucose as a carbon source. The lowest concentration of biomass was found in culture media supplemented with sugars not assimilated by *Saccharomyces* yeast, such as lactose, arabinose, xylose [17]. Inositol as a carbon source made it possible to achieve a maximum biomass concentration of 0.5 mg mL^−1^; the largest increase was observed between 48 and 72 h of culture (increase of over 0.3 mg mL^−1^; Figure 1A). Evaluation of the effect of carbon source on phytic acid hydrolysis showed no correlation between high yeast biomass concentration in culture media and high phytate utilization. After 72 h of cultivation in a medium supplemented with maltose with the highest concentration of biomass, the concentration of phytic acid was reduced by only ca. 25%, relative to the initial concentration (Figure 2A). The highest degree of hydrolysis of phytates (ca. 50%) was observed for media with mannose; however, a reduction of more than 35% relative to the initial concentration was also found in media supplemented with sucrose, fructose and glucose (Figure 2A). The lowest level of phytate utilization not exceeding 4% was observed in the media with the lowest biomass concentration, i.e., in the media supplemented with lactose, arabinose, xylose and inositol (Figure 2A). Due to the highest level of phytic acid reduction in media with mannose, this carbohydrate was used in further research as the carbon source.

**Figure 3:**
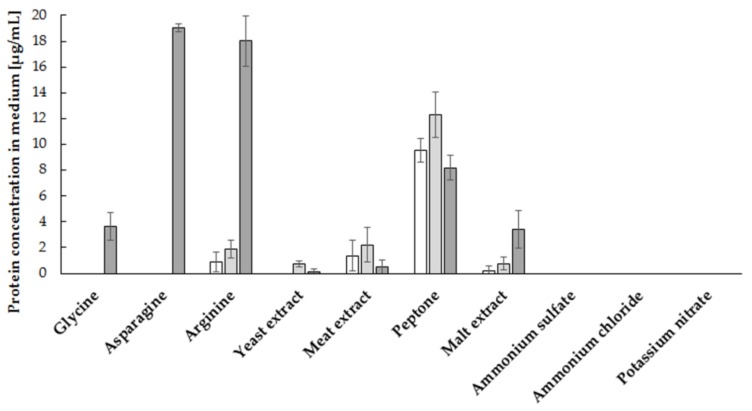
Influence of nitrogen source on extracellular protein concentration during culture of *S. cerevisiae* strain Finarome (24 h of culture—white bars, 48 h of culture—light gray bars, 72 h of dark gray bars).

Analysis of the influence of various sources of nitrogen on the growth of *S. cerevisiae* (strain Finarome) biomass confirmed that the best source of nitrogen is amine nitrogen derived not only from various types of hydrolysates (yeast, malt, beef extracts and peptone) but also from single amino acids (glycine, arginine, asparagine). For culture media with a complex mixture of amino acids and other biologically active compounds (mostly B vitamins) derived from yeast, beef extract and peptone, the highest biomass concentration (more than 6 mg mL^−1^) was observed after 72 h of culture (Figure 1B). The use of individual amino acids resulted in a maximum biomass concentration of more than 1.5 mg mL^−1^, while the addition of mineral nitrogen (ammonium, nitrate) caused an increase in yeast biomass of up to 1 mg mL^−1^ (Figure 1B). The use of amine nitrogen led to the reduction of phytate concentration by more than 80% after 72 h of culture, compared to the initial concentration. The application of arginine or asparagine resulted in the complete utilization of phytic acid after 48 h of culture. Supplementation with asparagine caused a reduction of phytic acid concentration by more than 85% after 24 h (Figure 2B). Moreover, the addition of asparagine to the culture media gave the highest concentration of extracellular proteins in the media, above 19 μg mL^−1^ (Figure 3). A significant reduction in phytic acid concentrations in asparagine-supplemented media was the reason why we used this compound as the source of nitrogen in subsequent stages of the selection of culture conditions for the production of phytases using *S. cerevisiae,* strain Finarome.

The effect of pH on yeast biomass growth during culture and on phytate reduction was also evaluated. The highest biomass concentration (greater than 6 mg mL^−1^) was found at pH 5.5 (Figure 1C). The increase in yeast biomass was slow in the first 48 h of culture at pH 3.5, 4.5 and 6.5 and below 2 mg mL^−1^. However, irrespective of the pH value of the culture medium, the largest increase in yeast biomass was observed between 48 and 72 h of culture (Figure 1C). The highest reduction in phytic acid concentration (of more than 90%) was also observed at this pH value after 24 h of culture (Figure 2C). Therefore, at this pH a further parameter was analyzed, i.e., the influence of temperature on biomass growth and the level of utilization of phytates. The highest concentration of biomass after 72 h of culture, 6 mg mL^−1^, was found at 28 °C, but the highest level of utilization of phytic acid (over 98%) after 24 h of culture was reached at 32 °C (Figure 1D and Figure 2D). Temperature lower (24 °C) or higher (32 and 37 °C) than the most convenient one resulted in a low biomass concentration, which was below 2 mg mL^−1^ even after 72 h of culture (Figure 1D).

**Figure 4:**
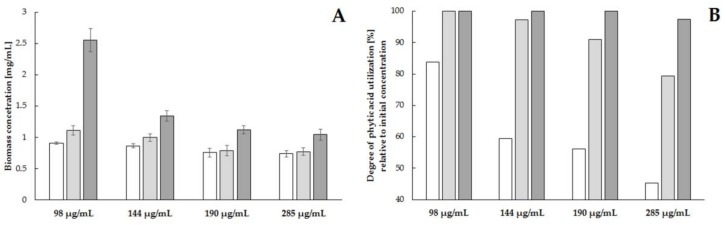
Effect of phytic acid concentration on growth rate of yeast biomass (**A**) and degree of reduction of phytic acid concentration (**B**) during culture of *S. cerevisiae* strain Finarome (24 h of culture—white bars, 48 h of culture—light gray bars, 72 h of dark gray bars).

The selection of the media composition and the culture conditions allowed us to assess the suitability of *S. cerevisiae*, strain Finarome, for the utilization of phytic acid during culture on media with elevated content of phytates. Using different concentrations of phytic acid (98, 144, 190, 285 µg mL^−1^) as the primary source of phosphorus, we observed the highest concentration of yeast biomass (2.5 mg mL^−1^) when the concentration of phytic acid in the medium was 98 μg mL^−1^. In the media with a higher phytic acid concentration (144, 190, 285 µg mL^−1^), the maximum biomass content did not exceed 1.5 mg mL^−1^ (Figure 4A). However, the effective selection of yeast culture (the use of appropriate carbon and nitrogen sources at the selected temperature and pH) allowed the phytic acid concentration to be reduced by more than 97% during 72 h of culture with an initial concentration of 285 μg/mL, which is nearly three times higher than in earlier studies (Figure 4B). The ability of the Finarome strain to utilize phytic acid at such a high concentration makes the strain useful for further studies on electrofusion (for example with *S. cerevisiae* distiller’s strain) to obtain a fusant that would efficiently hydrolyze phytates during alcoholic fermentation of HG (High Gravity) and VHG (Very High Gravity) media.

Bacteria, fungi and yeast require different nutrients for proper growth and enzyme biosynthesis. There are differences in the preferred composition of culture media between genera, species and even strains of microorganisms. It is essential to choose a suitable source of carbon, nitrogen and their relative proportions [8]. In the studies conducted on CY strain of *S. cerevisiae* [14] it was shown, as in the present work, that inositol and sugars not assimilated by yeasts (arabinose, xylose, lactose) were not suitable for the production of phytase. It was also found, as in our own studies, that fructose, glucose, galactose, mannose, maltose and sucrose had a positive effect on yeast biomass growth. In, et al. [14] also observed that phytases isolated from media supplemented with fructose, glucose and galactose (as the primary source of carbon), exhibited an elevated activity. Bogar, et al. [7] reported a positive effects of starch and sucrose from molasses on phytase production in their studies conducted with NRRL 3135 strain of *Aspergillus ficuum.* Optimization of *Aspergillus tubingensis* culture conditions demonstrated that glucose and sucrose were carbon sources for which the highest phytase activity was observed. This is also confirmed by our research indicating that these sugars are one of the better choices for *S. cerevisiae* yeast cultures focused on the production of phytases. However, in the case of *A. tubingensis*, the most effective source of nitrogen, which guaranteed a high phytase productivity, was mineral nitrogen in the form of ammonium sulfate and ammonium nitrate. This fungus and *S. cerevisiae* strain Finarome have similar culture conditions for phytase production: temperature 30 °C and pH 5–5.5 [18]. The application of different nitrogen sources in *Pichia anomala* culture focused on phytase production was analyzed by Kaur and Satyanarayana [9]. These studies also showed asparagine as a nitrogen source to achieve the highest yield in phytase production. Asparagine was also shown to stimulate phytase production in studies using *Sporotrichum thermophile* [12]. Research on the CY *S. cerevisiae* strain demonstrated that the optimum mineral nitrogen source for phytase production was ammonium sulfate. This shows how large differences in the optimal source of nitrogen are between the different strains of microorganisms [14]. The influence of temperature and pH on phytase production by *Pichia anomala* was investigated by Vohra and Satyanarayana [19]. It was also reported that the optimum temperature for yeast phytase was 25 °C, and optimum pH was 6. Due to the large metabolic differences between the different strains, detailed studies on optimal nutrients and culture conditions are needed to obtain a high degree of utilization of phytic acid. This is confirmed by our own studies supported by the analysis of available literature.

### 2.2. Catalytic Characteristics of Phytase Isolated from S. cerevisiae Strain Finarome

Preliminary characterization of catalytic activity of enzymes isolated from microorganism cultures is an important step in the study aimed to evaluate the practical use of biocatalysts. Considering the use of phytase isolated from *S. cerevisiae* yeast strain Finarome for the hydrolysis of phytates during the efficient fermentation process, we analyzed not only the basic phytase properties (temperature and pH, the effect of metal ions on catalytic activity), but also the effect of ethanol and substrate concentration on the activity of this enzyme. The obtained characteristics of the biocatalyst may be useful in deciding whether to use the subject strain for electrofusion with an efficient ethanol-producing strain.

**Figure 5:**
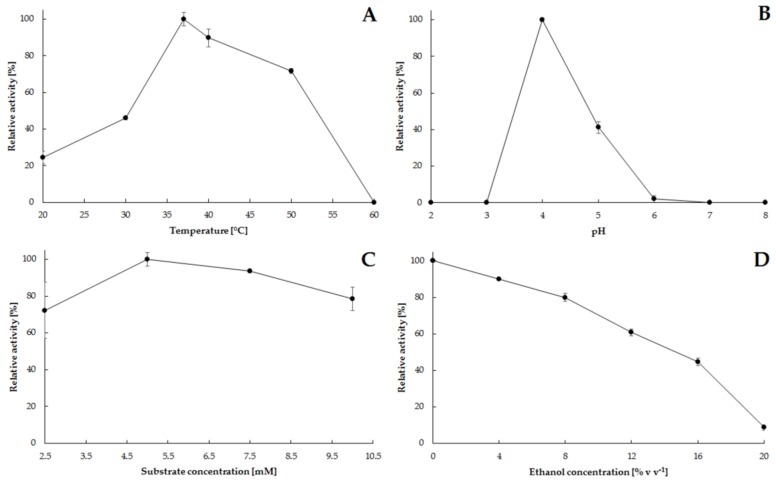
Evaluation of the influence of temperature (**A**), pH (**B**), substrate (phytic acid) concentration, (**C**) and ethanol concentration (**D**) on the activity of phytase isolated from *S. cerevisiae* yeast strain Finarome.

Analysis of the influence of basic parameters characterizing enzymatic proteins revealed that the highest catalytic activity was obtained for phytase isolated from the wine yeast culture at 37 °C. This temperature is optimal for conducting alcoholic fermentation using most distillery yeast strains (Figure 5A) [20]. Considering the technological parameters of industrial ethanol production, it is important to confirm the high activity of the analyzed phytase at elevated temperatures. At 40 °C, 90% of the maximum activity was reached; at 50 °C the percentage was about 72% (Figure 5A). In addition, maintaining high activity of phytase isolated from the Finarome strain at elevated temperatures makes it possible to use this enzyme in feed production. The increased thermostability is most probably a result of post-translational glycosylation in yeast expression systems [15,21].

Isolated phytase had a relatively narrow range of pH, where higher catalytic activity was observed. Maximum activity was observed at pH 4 (Figure 5B). High catalytic activity in this pH range indicates that there is potential for the use of the strain Finarome as a phytase producer when attempting to obtain a fusant yeast capable of utilizing phytate in alcoholic fermentation, most commonly carried out in the pH range of 4.0 to 5.5 [20]. The influence of selected metal ions (as activators or inhibitors) on yeast phytase activity was also evaluated. A reduction of phytase catalytic activity was observed for silver, mercury and copper salts (5 mM), but the addition of Ag^2+^ and Hg^2+^ resulted in an almost complete inhibition of phytic acid hydrolysis (Table 1). Supplementation of the reaction medium with the remaining metal ions resulted in an increase in the catalytic activity of the phytase relative to the enzyme solution without the addition of metals. The highest increase in activity (more than 20%) was observed when magnesium, manganese and nickel salts were added at a concentration of 5 mM (Table 1). The use of higher concentrations of metal salts (10 mM) resulted in a further decrease in phytase activity as compared to the 5 mM concentration. This was not the case for calcium ions added at elevated concentrations, which maintained an elevated catalytic activity of approximately 112% (Table 1). The effects of the addition of selected metal ions to the systems formed of two metal salts were also analyzed. Their addition resulted in a significant increase in phytase activity at a salt concentration of 5 mM (Table 2). However, statistical analysis did not reveal any statistically significant differences between the studied metal systems and the enzyme solution without the addition of metal ions. This may be due to the phenomenon of ionic antagonism that suppresses the positive effect of selected metal ions on the activity of isolated phytase. In order to assess the possibility of using the strain Finarome for the utilization of phytate during fermentation processes in media containing elevated phytic acid, the effects of substrate and ethanol concentrations on phytase activity were also studied. Our studies showed a high catalytic activity of phytase for increasing substrate concentration. The highest activity was observed at phytic acid concentration of 5 mM. However, the use of a concentration in the range of 2.5 to 10.0 mM reduced the catalytic activity of phytase to a maximum of 72% (Figure 5C). An increasing ethanol concentration during the reaction catalyzed by phytase isolated from the Finarome strain reduced the activity to a maximum of ca. 8% for 20% (*v*/*v*) ethanol (Figure 5D). The application of ethanol concentration of 8–12% *v*/*v* in the first two days of alcoholic fermentation using HG media [22], resulted in a decrease of activity to about 70% (Figure 5D). This indicates the usefulness of this yeast strain in the research on obtaining a highly efficient fusant capable of utilizing phytic acid during the alcoholic fermentation.

In the case of phytase isolated from other groups of microorganisms such as bacteria and filamentous fungi, optimum temperature and pH were observed in the range higher than in the presented studies on yeast phytase. Phytases isolated from various bacterial strains of the genus *Bifidobacterium* carry out the hydrolysis of phytic acid at a pH of 5 to 8, whereas the fungal phytase from the *Aspergillus* genus is most efficient at a pH between 5 and 7 [15,23,24]. When comparing the catalytic properties of the analyzed phytase with another phytase isolated from *S. cerevisiae* yeast culture, some similarities can be noted. In et al. [25] also analyzed the activity of yeast phytase. Similarly to our study, a relatively narrow range of pH in the range of 3.5–4.0 and a lower catalytic activity of phytase above 40 °C were observed. The most convenient temperature for hydrolysis of phytic acid for phytase isolated from *S. cerevisiae* strain CY was 35–40 °C. Phytase activity drastically decreased above 45 °C and was only about 10% of the maximum catalytic activity at 50 °C. Furthermore, the phytase isolated from this wine yeast strain was characterized by a much lower resistance to the presence of metal cations in the medium. The addition of most of them (CaCl_2_, CoCl_2_, CuCl, FeCl_2_, MgCl_2_, MnCl_2_, NiCl_2_) reduced the catalytic activity of the phytase even to a level of 20% of maximum activity [25]. The effect of the metal compounds on the activity of phytase isolated from *Kodamaea ohmeri* sea yeast and the *Aspergillus niger* fungi was different. Analysis of the effect of metal ions on phytase activity isolated from the yeast culture of *Kodamaea ohmeri* revealed relationships similar to those shown in our studies. The addition of 5 mM magnesium, cobalt, manganese and calcium compounds increased the catalytic activity of the phytase by more than 10% compared to the reaction without the addition of metal cations [26]. Studies on the ATCC 9142 strain of *Aspergillus niger* confirmed the possibility of increased phytase activity in the presence of selected metal ions, but it should be noted that this may be an individual characteristic of the selected strains of microorganisms. The increase in the concentration of magnesium, manganese, cadmium and zinc compounds increased the catalytic activity of phytase isolated from the ATCC 9142 strain of *A. niger*, as in our research. In the studies with *A. niger*, this increase was more than 42% in relation to maximum activity in the medium without the addition of metal ions, while in our studies of *S. cerevisiae* strain Finarome it amounted to at most 25% [27].

## 3. Materials and Methods

### 3.1. Yeast Strain and Inoculum Preparation

In our experiments we used a preparation of dried *S. cerevisiae* yeast, wine strain Finarome, by Prédel Oenologie (Montussan, France). This strain, isolated in Alsace, is used for the production of white wines of the Muscat and Riesling types. It has a high nitrogen demand and exhibits a high capacity of phytic acid utilization in aerobic cultures [16]. The yeast inoculum was prepared by suspending 1 g of dried yeast in 10 mL of sterile 0.9% NaCl and rehydrating it for 10 min. 100 mL of the culture medium was inoculated with 0.1 mL of yeast milk (1.03 ± 0.05 × 10^9^ CFU mL^−1^).

### 3.2. Culture Conditions Used for the Selection of the Composition of the Culture Media

Cultures were grown in 250 mL Erlenmeyer flasks containing 100 mL of medium with the alternating carbon source (carbon source 10 g L^−1^: sucrose, maltose, fructose, galactose, arabinose, mannose, glucose, xylose and inositol; (NH_4_)_2_SO_4_ 3 g L^−1^; MgSO_4_ 0.5 g L^−1^; KCl 0.Yeast were 5 g L^−1^; FeSO_4_ 0.001 g L^−1^; MnSO_4_ 0.0075 g L^−1^; CaCl_2_ 0.1 g L^−1^; phytic acid 0.1 g L^−1^), at 28 °C and pH 5.5 for 72 h with continuous shaking at 150 rpm. Selection of a nitrogen source was carried out using a medium containing mannose (10 g L^−1^) as a carbon source and various nitrogen sources (10 g L^−1^: glycine, asparagine, arginine, yeast extract, bovine extract, peptone, malt extract, (NH_4_)_2_SO_4_, NH_4_Cl_2_, KNO_3_). The medium contained also: MgSO_4_ 0.5 g L^−1^; KCl 0.5 g L^−1^; FeSO_4_ 0.001 g L^−1^; MnSO_4_ 0.0075 g L^−1^; CaCl_2_ 0.1 g L^−1^; phytic acid 0.1 g L^−1^. Cultures were run at 28 °C and pH 5.5 for 72 h. The influence of culture conditions, i.e., temperature (24, 28, 32, 37 °C), pH (3.5, 4.5, 5.5, 6.5) and the substrate concentration (98, 144, 190, 285 μg mL^−1^) on the utilization rate of phytic acid was tested on a medium of the following composition: mannose 10 g L^−1^; asparagine 10 g L^−1^; MgSO_4_ 0.5 g L^−1^; KCl 0.5 g L^−1^; FeSO_4_ 0.001 g L^−1^; MnSO_4_ 0.0075 g L^−1^; CaCl_2_ 0.1 g L^−1^; phytic acid 0.1 g L^−1^ [28].

### 3.3. Culture Conditions Used to Determine Catalytic Activity of Phytase 

In order to obtain the enzyme, yeast inoculum (0.1 mL of yeast milk) was added to a culture medium (100 mL) containing: mannose 10 g L^−1^; asparagine 10 g L^−1^; MgSO_4_ 0.5 g L^−1^; KCl 0.5 g L^−1^; FeSO_4_ 0.001 g L^−1^; MnSO_4_ 0.0075 g L^−1^; CaCl_2_ 0.1 g L^−1^; phytic acid 0.1 g L^−1^. The cultures were kept at 32 °C, pH 5.5, for 72 h with continuous shaking at 150 rpm.

### 3.4. Analytical Methods

During the selection of the culture medium composition aimed at phytase production, the concentration of biomass, phytic acid and extracellular proteins was analyzed at 24, 48, and 72 h of culture. During the culture, the reduction of phytic acid concentration (%) was calculated as compared to the initial concentration. Yeast biomass was analyzed based on optical density (measured with Pharo 300, Merck^®^) at OD_600_ versus calibration curve prepared for *S. cerevisiae* yeast cells [29]. The concentration of phytic acid in the culture medium was determined after separation of yeast cells from the medium using membrane filters, 0.45 μm pore size (Agilent Technologies^®^, Santa Clara, CA, USA). Phytic acid was determined by spectrophotometric method at 510 nm on Pharo 300 apparatus (by Merck^®^, Darmstadt, Germany) using WADE reagent (0.27 g FeCl_3_ × 6H_2_O, 2.54 g C_7_H_6_O_6_S × 2H_2_O), with sodium phytate (C_6_H_6_O_24_P_6_Na_12_) as the standard [30]. The concentration of extracellular protein was determined in a yeast-free media by Bradford’s spectrophotometric method using Pharo 300 (by Merck^®^) against the calibration curve for bovine albumin [31].

### 3.5. Determination of Catalytic Activity of Phytase

To isolate yeast cells, the medium was centrifuged (at 5000 rpm, 15 min, 4 °C) after 72 h of culture. The post-culture solution was then concentrated by ultrafiltration with the use of Amicon Ultra 10,000 MWCO system (Merck-Millipore^®^, Darmstadt, Germany) and centrifugation at 500 rpm for 3 h at 4 °C. The resulting protein concentration was about 200.0 ± 2.7 µg mL^−1^. The protein solution was stored at −20 °C until catalytic activity was determined. We analyzed the influence of temperature (20, 30, 37, 40, 50, 60 °C), pH (2, 3, 4, 5, 6, 7, 8) (pH buffers: 2, 3—0.1 M glycine and 0.1 M HCl; 4, 5, 6—0.1 M acetate buffer; 7, 8—0.1 M Tris-HCl buffer), sodium phytate concentration (2.5; 5.0; 7.5; 10.0 mM), ethanol concentration (0; 4; 8; 16; 20% *v*/*v*), and selected metal ions (Zn^2+^, K^+^, Ca^2+^, Cu^+^, Mg^2+^, Mn^2+^, Fe^3+^, Ag^2+^, Ni^2+^, Co^2+^, Hg^2+^, Cd^2+^) (Zn^2+^, Mg^2+^, Mn^2+^, Fe^3+^, Ag^2+^, Hg^2+^, Cd^2+^—sulphates; K^+^, Ca^2+^, Cu^+^, Ni^2+^, Co^2+^—chlorides) in 5 and 10 mM concentrations on the catalytic activity of phytase isolated from cultures of *S. cerevisiae*, strain Finarome. Evaluation of catalytic activity of phytase was carried out using Shimizu method with modifications [32]. The concentrated protein solution was first diluted in 0.2 M acetate buffer at pH 5.0. A solution of enzyme proteins (0.2 mL) was incubated for 5 min at 37 °C. The same procedure was used for the blank and standard sample (0.2 mL 0.2 M acetate buffer pH 5.0), but trichloroacetic acid (1.0 mL) was also added to these tests. Subsequently, a solution of the substrate (0.8 mL of sodium phytate solution in 0.2 M acetate buffer at pH 5.0) was added to each sample. The samples were then mixed and incubated for 30 min at 37 °C. A solution of trichloroacetic acid (1.0 mL) was added to the experimental samples to stop the enzymatic reaction. In order to determine the concentration of orthophosphate, a colored solution was added. The solution was obtained by mixing solutions A and B in a 4:1 ratio; solution A: 7.5 g of ammonium heptamolybdate (N_6_H_24_Mo_7_O_24_ × 4 H_2_O), 22 mL of sulfuric acid (98%) in 500 mL of the solution; solution B: 2.7% iron sulfate solution. All samples were centrifuged (at 5000 rpm, 20 °C, for 10 min). Spectrophotometric measurements of orthophosphate concentrations were performed (with Pharo 300, Merck^®^) at λ = 700 nm against the standard curve for KH_2_PO_4_. A unit of phytase activity was defined as the amount of enzyme that liberates 1 µmol P min^−1^ µg of protein^−1^ under the given assay conditions.

### 3.6. Statistical Analysis

All analyses and cultures were done in triplicate. The results obtained were subjected to statistical analysis (average values, standard deviations, ANOVA module, variance analysis at α < 0.05) using Statistica software, version 12.

## 4. Conclusions

The obtained results confirm that the *S. cerevisiae* yeast culture aimed at efficient production of extracellular phytases requires the development of a biosynthesis process to select both the composition of the culture medium and the culture conditions. The most effective for the Finarome strain was the application of mannose as a source of carbon, asparagine as a source of nitrogen, temperature 32 °C and pH 5.5. Under these conditions, the concentration of phytic acid added to the medium at 98 μg/mL was reduced by 98% in the first 24 h of culture. When the concentration of phytic acid was elevated to 285 μg mL^−1^, it was possible to achieve almost complete utilization but in a process extended to 72 h. During the selection of culture conditions, no correlation was found between the rate of phytic acid utilization and the yeast biomass concentration when selecting the source of carbon, nitrogen and the temperature of the culture. Only at pH 5.5 the highest biomass concentration and the lowest concentration of phytic acid were observed. In addition, we confirmed the effectiveness of the OFAT method (one-factor-at-a-time method) in the selection of the source of carbon, nitrogen, pH level and temperature of culture that would guarantee full utilization of phytic acid during 72 h of Finarome culture. The phytase isolated after culture of the Finarome strain exhibited a maximum catalytic activity at 37 °C and pH 4.0. The isolated enzyme exhibited a high activity (about 70%) at ethanol concentration of 8–12% *v*/*v*. The presence of magnesium, manganese and nickel ions at a concentration of 5 mM resulted in an increase in the phytase activity by more than 20%. An elevated resistance of the isolated phytase to zinc, copper, iron and cadmium ions (which decrease the enzyme activity) was observed. Only a high concentration of these ions (10 mM) resulted in a decrease in the catalytic activity of the enzyme, from 6 to 39%. The catalytic properties of phytase isolated from *S. cerevisiae* yeast, strain Finarome, suggest the possibility of its practical use for phytic acid hydrolysis during highly efficient alcoholic fermentation processes using HG or VHG media. It is worthwhile considering the use of Finarome strain to obtain a fusant, capable of utilizing phytate in fermentation processes, via electrofusion with an efficient osmotolerant and alcohol tolerant distillery strain.

## Figures and Tables

**Table 1:** Effect of addition of metal ions on catalytic activity of phytase isolated from *S. cerevisiae* Finarome strain.

Reagents	Relative Activity (%)	
	5 mM	10 mM
Control	100.0 ± 1.3	100.0 ± 0.5
ZnSO_4_	113.0 ± 0.6	84.7 ± 0.3
KCl	104.7 ± 5.6	102.6 ± 2.3
CaCl_2_	112.0 ± 1.4	111.8 ± 0.3
CuCl_2_	91.6 ± 1.1	61.4 ± 0.4
MgSO_4_	125.0 ± 1.3	106.1 ± 0.7
MnSO_4_	120.8 ± 2.0	106.7 ± 1.3
FeSO_4_	112.6 ± 1.3	93.8 ± 0.0
AgSO_4_	0.0 ± 0.0	0.0 ± 0.0
NiCl_2_	125.1 ± 0.0	105.3 ± 0.5
CoCl_2_	108.5 ± 0.4	106.7 ± 1.6
HgSO_4_	11.7 ± 7.3	0.0 ± 0.0
CdSO_4_	109.2 ± 1.4	88.0 ± 0.6

Before the activity measurement, the enzyme was incubated for 20 min in a 5 and 10 mM solution of metal ions. Activity measurements were performed under the following conditions: substrate concentration of 5 mM, temperature 37 °C, pH 4.

**Table 2:** Effect of addition of 5 mM metal ion mixtures on catalytic activity of extracellular phytase isolated from *S. cerevisiae* Finarome strain.

Reagents	Relative Activity (%)
	5 mM
Control	100.0a ± 1.1
CaCl_2_ + MnSO_4_	97.1a ± 1.1
CaCl_2_ + NiCl_2_	100.6a ± 1.5
CaCl_2_ + MgSO_4_	98.3a ± 1.1
CaCl_2_ + CoCl_2_	100.3a ± 0.2
MnSO_4_ + NiCl_2_	99.2a ± 1.0
MnSO_4_ + MgSO_4_	97.3a ± 1.3
MnSO_4_ + CoCl_2_	98.3a ± 1.1
MgSO_4_ + NiCl_2_	98.9a ± 0.8
MgSO_4_ + CoCl_2_	97.4a ± 2.5
NiCl_2_ + CoCl_2_	100.3a ± 0.6

The mean values given in columns with different letter index are significantly different (a < 0.05). Before the activity measurement, the enzyme was incubated for 20 min in a 5 mM solution of metal ions. Activity measurements were performed under the following conditions: substrate concentration of 5 mM, temperature 37 °C, pH 4.
